# OSBP-Related Proteins: Liganding by Glycerophospholipids Opens New Insight into Their Function

**DOI:** 10.3390/molecules181113666

**Published:** 2013-11-05

**Authors:** Vesa M. Olkkonen

**Affiliations:** 1 Minerva Foundation Institute for Medical Research, Biomedicum 2U, Tukholmankatu 8, Helsinki FI-00290, Finland; E-Mail: vesa.olkkonen@helsinki.fi; Tel.: +35-89-1912-5705; Fax: +35-89-1912-5701; 2 Institute of Biomedicine, Anatomy, Biomedicum 1, Haartmaninkatu 8, University of Helsinki, Helsinki FI-00014, Finland

**Keywords:** cell signaling, cholesterol, lipid metabolism, LTP, ORP, OSBPL, Osh, oxysterol, phospholipid

## Abstract

Oxysterol-binding protein (OSBP) and its homologs designated OSBP-related (ORP) or OSBP-like (OSBPL) proteins constitute a conserved family of lipid binding/transfer proteins (LTP) in eukaryotes. The mechanisms of ORP function have remained incompletely understood, but they have been implicated as intracellular sterol sensors or transporters. A number of studies have provided evidence for the roles of ORPs at membrane contact sites (MCS), where endoplasmic reticulum is closely apposed with other organelle limiting membranes. ORPs are postulated to either transport sterols over MCSs or control the activity of enzymatic effectors or assembly of protein complexes with functions in signaling and lipid metabolism. Studies of yeast *Saccharomyces cerevisiae* ORPs Osh4p, Osh3p, Osh6p and Osh7p have revealed that ORPs do not exclusively bind sterols within their OSBP-related ligand-binding domain (ORD): The Osh4p ORD accommodates either sterols or phosphatidylinositol-4-phosphate (PI4P), and the Osh3p ORD was shown to specifically bind PI4P, the binding cavity being too narrow for a sterol to fit in. Most recently, Osh6p and Osh7p were demonstrated to show specific affinity for phosphatidylserine (PS), and to play a role in the intracellular transport of this glycerophospholipid; Additionally, two mammalian ORPs were shown to bind PS. Thus, the term frequently used for ORPs/OSBPLs, *oxysterol-binding proteins*, is a misnomer. While a number of ORPs bind oxysterols or cholesterol, other family members appear to interact with phospholipid ligands to regulate lipid fluxes, organelle lipid compositions and cell signaling. As a conclusion, ORPs are LTPs with a wide ligand spectrum and marked functional heterogeneity.

## 1. Introduction

The compartmentalization of lipid synthetic processes and the inter-organelle distributions of lipids in eukaryotic cells necessitate well controlled lipid fluxes [1]. The intracellular transport of lipids is mediated by four major mechanisms: (i) Flip-flop from one leaflet of a bilayer to the other [2,3]; (ii) movement as components of transport vesicles/tubular carriers [4,5]; (iii) diffusion along membrane bilayers and between closely apposed membrane leaflets [1], (iv) transfer by lipid binding/transfer proteins (LTPs) [6,7]. The latter two mechanisms represent non-vesicular inter-bilayer lipid transport and play important roles in lipid fluxes between organelles not connected via the established pathways of membrane trafficking. The markedly hydrophobic nature of most membrane lipids makes their diffusion through the aqueous cytosolic phase energetically unfavourable. Therefore, non-vesicular lipid transfer necessitates either close proximity of the two membranes and/or the involvement of LTPs that carry a bound lipid across the aqueous phase [7]. Membrane contact sites (MCS) are zones of close apposition (10–30 nm distance) of endoplasmic reticulum (ER) membranes with other organelle limiting membranes. These contact sites have established or postulated roles in lipid syntheses, inter-organelle lipid transport, Ca^2+^ signaling, and ER function [8,9,10,11].

Cholesterol is the most abundant single lipid molecular species in mammalian cells. It plays a central role in controlling the fluidity, permeability, and microdomain organization of specific membrane bilayers, as well as the activity of a number of membrane-associated proteins [12]. Cholesterol displays an uneven distribution in cellular membranes, being enriched towards late Golgi and the PM, as well as in endosomal compartments. The mechanisms maintaining this asymmetry are not perfectly understood, but partitioning of cholesterol into laterally organized domains together with sphingolipids and glycerophospholipids with a high degree of fatty acyl chain saturation is suggested to play an important role [13,14]. Cholesterol is markedly hydrophobic, and even though part of it is transported within cells along with vesicular/tubular carriers of the vesicle transport pathways, its efficient inter-organelle transport needs to be mediated to a large extent by non-vesicular processes facilitated e.g., by LTPs and/or closely apposed membrane domains, similar to the transport of glycero- and sphingolipids with physiologic, long-chain fatty acyl chains [7,15,16]. Oxysterols, 27-carbon oxygenated derivatives of cholesterol, are formed by enzymatic or non-enzymatic oxidative processes [17,18], and are present in healthy tissues in trace amounts as compared to cholesterol [19,20]. Oxysterols differ quite significantly from cholesterol in their biophysical properties, such as their orientation in bilayers, impacts on membrane lipid packing as well as their increased solubility and high rate of spontaneous inter-membrane transport [21,22,23,24]. The latter property prompts the question of whether carrier proteins are necessary for the inter-organelle transport of oxysterols, or whether spontaneous transfer is sufficient to account for the observed rapid movement. When accumulating in pathologic tissues, oxysterols display adverse, cytotoxic effects [25,26,27,28,29]; However, a number of endogenous cellular oxysterols, especially those modified by hydroxylation of the sterol side chain, have crucial signaling functions via liganding of transcription factors and protein regulators of metabolism [20,30].

## 2. OSBP-Related Proteins and Their Liganding by Sterols

**Figure 1:**
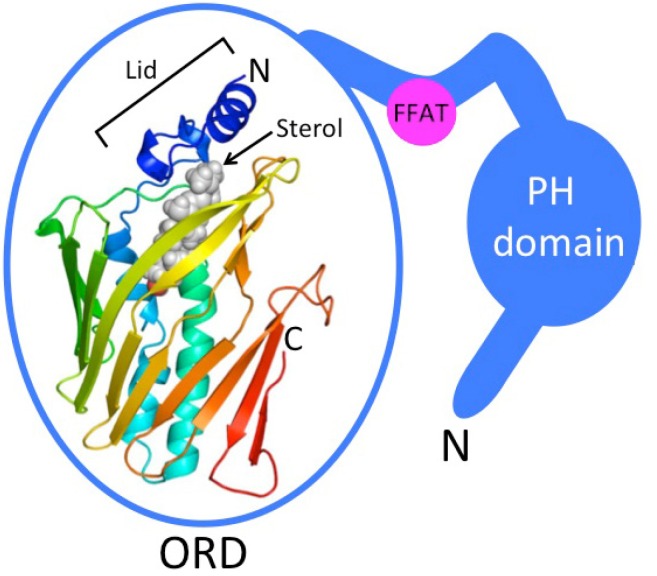
Schematic structure of ORP proteins. ORD, OSBP-related ligand binding domain, represents human ORP1 ORD modeled by using the yeast Osh4p structure [33] as a template. Bound sterol, which stabilizes a closed conformation of a flexible ‘Lid’ structure, is depicted as a gray ball-model. β-strands are displayed as flat arrows and α-helical secondary structures as helices. While “Short” subtype ORPs, such as yeast Osh4p-Osh7p, consist of a mere ORD, the “Long” subtype family members (most mammalian ORPs belong to this category) carry an N-terminal extension (blue) with a pleckstrin homology (PH) domain interacting with phosphoinositides and a short peptide motif (two phenylalanines in an acidic tract, FFAT) that targets the ER.

Oxysterol-binding protein family members, designated OSBP-related (ORP) or OSBP-like (OSBPL) proteins, are LTPs characterized by a carboxy-terminal OSBP-related ligand-binding (ORD) domain; In addition, most of them have a pleckstrin homology (PH) domain in their N-terminal part, involved in membrane targeting of the proteins, and either a short amino acid motif (FFAT, two phenylalanines in an acidic tract) or a C-terminal trans-membrane span specifying association of the proteins with ER membranes [31,32,33,34,35] (Figure 1). In mammals the protein family is encoded by 12 genes [31,34,35], while in yeast *Saccharomyces cerevisiae* there are seven ORP genes/proteins designated OSBP homologs (Osh; [32]). The ORDs of a number of ORPs were found to accommodate a variety of oxysterols, cholesterol, or ergosterol [33,36]. The hallmark study of Im *et al.* [33] reported the high-resolution structure of yeast Osh4p complexed with five different sterols. This study revealed a β-barrel like fold with a lipid-binding cavity, in which the bound sterol is oriented with its 3β-hydroxyl group facing the bottom of the pocket (Figure 1).

Bound sterol stabilizes a closed conformation of a lid composed of a 2-stranded β-sheet and three α-helices, which makes contacts with the sterol iso-octyl side chain. Non-sterol bound Osh4p could not be crystallized, apparently due to an unorganized state of the lid. The lid-open Osh4p was suggested to expose basic amino acid residues near the mouth of the ligand cavity that interact with phosphate groups at membrane surfaces, thus facilitating sterol extraction from the bilayer. Studies in yeast have provided evidence for a function of Osh4p and other yeast ORPs in sterol transport from the PM to the ER [37,38]; However, contradicting findings rather suggesting function of the Osh proteins as modifiers of the lateral organization of sterol at the PM have been reported [39].

OSBP, the prototype mammalian ORP, binds 25-hydroxycholesterol (25OHC) with a K_d_ of 10 nM, the affinity for other oxysterols being lower. OSBP also binds cholesterol, with a K_d_ of 170 nM [40,41,42,43,44]. The other human ORPs thus far studied display different affinities for various oxysterols, with K_d_s in the nM-μM range [43,45,46], and like OSBP, several of them are also suggested to bind cholesterol [45,47,48]. Considering that the cellular concentration of cholesterol vastly exceeds that of oxysterols, one could argue that cholesterol may be a more relevant physiologic ligand of these proteins than oxysterols [33]. However, oxysterols are markedly more aqueous soluble than cholesterol, which may greatly increase their accessibility for binding into the ORD. While oxysterol binding *in vitro* by purified ORPs has been demonstrated for OSBP, ORP4 (subfamily I), ORP1, ORP2 (subfamily II), and *in vitro* sterol transfer activity shown for OSBP, ORP9L, and the ORD of ORP5 [49,50], as well as the yeast *S. cerevisiae* ORPs Osh2p, Osh4p, and Osh5p (and a weaker activity for Osh1p, Osh3p) [37,38], the ligand binding properties of most ORPs (including the human proteins) have not been studied in detail. A study employing photo-cross-linkable cholesterol and 25OHC reported a positive cross-linking signal for either or both of these sterols for a total of human 10 ORPs [46]. In this study it remained, however, to some extent unclear whether all signals were due to sterol insertion within the ORD binding cavity, or if some of them may reflect cross-linking of the sterol derivatives to the surface of proteins associated peripherally or integrally with cell membranes.

## 3. ORPs Can Bind Glycerophospholipids

The extraction of sterols by Osh4p was shown to be markedly enhanced by inclusion of phosphatidylinositol-4,5-bisphosphate (PIP_2_) in the donor vesicles. Moreover, PIP_2_ as well as another acidic glycerophospholipid, phosphatidylserine (PS), in the donors were found to facilitate the transport of cholesterol from one vesicle population to another [37]. Such transfer stimulation was interpreted to reflect interactions of Osh4p with the charged membrane surface, facilitating its membrane docking and extraction of sterol from the bilayer, as suggested by Im *et al*. [33]. Intriguingly, however, Osh4p was also able to extract PIP_2_ from vesicles and displayed significant inter-vesicle transfer of this lipid [37], suggesting that PIP_2_ could actually be extracted from the membrane and be fully or partially inserted into the ligand cavity within the Osh4p ORD. Further work by Schulz *et al.* [38] identified on the ORD of Osh4p two membrane binding surfaces, one near the mouth of the ligand binding cavity and another distally located. The authors provided evidence suggesting that Osh4p employs these interaction surfaces to associate with two membranes simultaneously to catalyze inter-membrane sterol transfer.

De Saint-Jean *et al.* [51] determined the structure of Osh4p crystallized with PI4P inserted in the ORD pocket, and showed that a bound sterol (dehydroergosterol, DHE) is readily exchanged for PI4P. The authors suggested, based on *in vitro* evidence, that the two lipids could be transported by Osh4p in opposite directions in cells, *i.e.* sterol from the ER to the *trans*-Golgi/PM and PI4P backwards. This attractive model could explain how an increasing sterol gradient from the ER to the PM [12,13,52] is generated and why *OSH4* disruption in yeast results in a phenotypic bypass of mutations in other genes reducing late secretory pathway PI4P levels [53,54].

Mousley *et al.* [55] reported that a sterol-binding deficient mutant *osh4/kes1*^Y97F^ characterized by Alfaro *et al.* [56] disturbs cell proliferation due to its enhanced PI4P-dependent association with *trans*-Golgi network (TGN) and endosomes, thus representing a gain-of-function. The data strongly suggested that binding of sterol by Osh4p has a negative regulatory role, detaching the protein from TGN/endosome membranes [55], rather than a sterol transport function. The Osh4p-mediated growth arrest was in part due to amino acid deficiencies caused by defects in the trafficking of amino acid permeases, suppression of gene expression driven by Gcn4, a transcriptional activator of the general amino acid control (GAAC) regulon, and disturbed target of rapamycin complex 1 (TORC1) signaling. The TGN/endosomal signal generated by Osh4p remained incompletely understood, but it could involve sphingolipid enrichment in TGN/endosome membranes, a hypothesis supported by the study of LeBlanc *et al.* [57] who observed sphingolipid deficiencies in Osh4p-depleted yeast cells. The above studies are incompatible with a simple function of Osh4p as a sterol and PI4P transporter, but rather suggest a complex role in TGN/endosomal PI4P/sphingolipid signaling, with impacts on multiple cellular functions.

**Figure 2:**
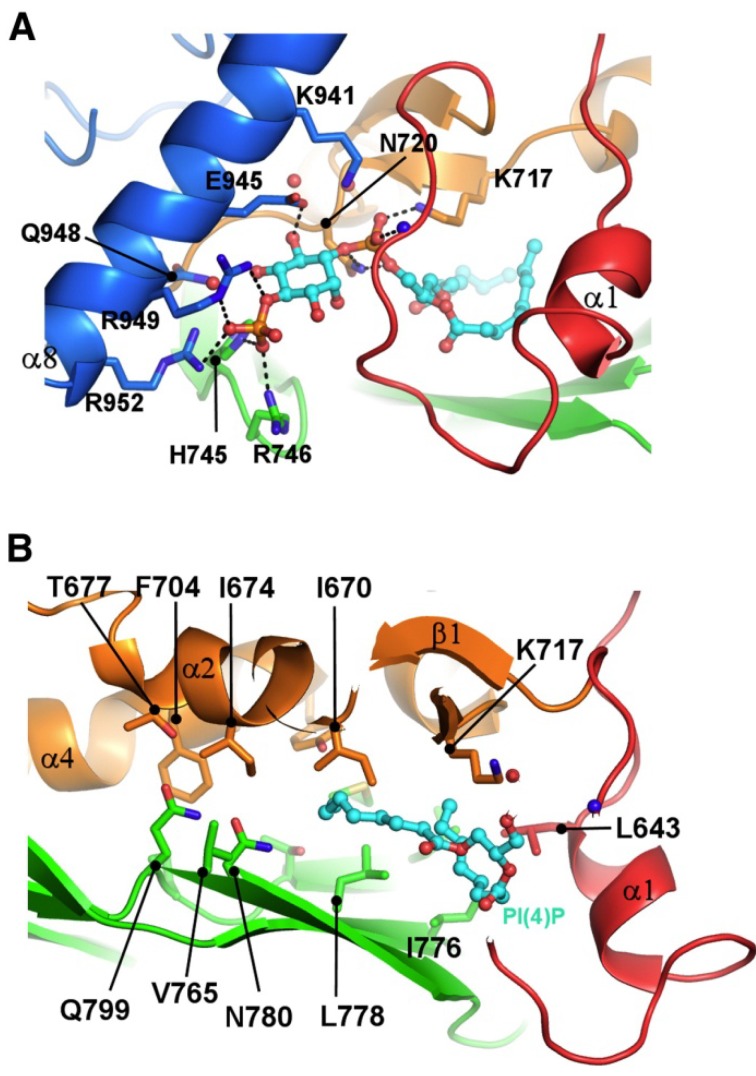
The yeast Osh3p ORD binds phosphatidylinositol-4-phosphate. (**A**) Binding of PI4P in the ORD. The two fatty acyl chains are inserted in the central hydrophobic tunnel, and the phosphoinositol head group is accommodated in a shallow basic cleft at the tunnel entrance. (**B**) The hydrophobic tunnel. The amino acid residues composing the wall of the tunnel are shown as ball-and-stick models. Reprinted with permission from Tong *et al*. [58] Copyright (2013) Elsevier Ltd.

Tong *et al.* [58] recently solved the structure of the Osh3p ORD with bound PI4P (Figure 2). The structure revealed that the ligand cavity is too narrow to accommodate bulky 4-ringed sterol molecules, strongly suggesting that Osh3p has no capacity of sterol binding. Of note, the authors pinpointed at the entrance of the ligand cavity a cleft that accommodates the phosphoinositol moiety of PI4P, and indicated that amino acid residues lining this cleft are highly conserved among all ORPs. This interesting observation, together with related findings by De Saint-Jean *et al.* [51], brought up the possibility that PI4P binding could be a unifying feature of all ORPs, and that only a subset of the family members could additionally be capable of accommodating sterol ligands.

The perception of ORP ligand specificity was further complicated by the latest report by Maeda *et al.* [59] showing that yeast Osh6p and Osh7p specifically bind PS, extract this phospholipid from membranes, and that disruption of *osh6/osh7* in yeast cells results in a defect of PS transport from the ER to the PM, as determined by imaging with a Lact C2-GFP probe. Osh6p was crystallized, and PS was modeled into the ORD ligand cavity: The head group and the unsaturated *sn-2* fatty acyl chain were oriented towards the entrance of the pocket, while a saturated *sn-1* chain pointed towards the bottom of the cavity. Interestingly, the functional defect in *osh6^−^/osh7^−^* cells appeared specific for the ER-PM transport step, since the authors, consistent with the earlier data of Raychaudhuri *et al*. [37], found no defect in conversion of PS to PE and PC, a process that involves non-vesicular transport of PS to mitochondria or the Golgi complex where PS decarboxylases are located [16]. Of note, Osh6p extracted no or very little ergosterol from membranes, suggesting that it, similar to Osh3p (see above), may be unable to bind sterols, thus putatively belonging to a new, PS-preferring ORP subgroup. In the light of these findings, the mechanisms resulting in the distortions in ergosterol metabolism in *OSH6* deletion or overexpressing yeast strains reported by Wang *et al.* [60] remain subject of further study.

Sequence analyses by Maeda *et al.* [59] suggested that among mammalian ORPs, ORP5, 8, 9, 10 and 11 belong to the same clade as Osh6p. Consistently, the ORDs of human ORP5 and 10 were shown to extract PS from liposomes. Some caution, however, is appropriate in interpreting *in vitro* lipid binding data obtained using fusion proteins produced in *E. coli*: Partial misfolding of recombinant proteins may expose regions with artefactual affinity for distinct lipids. The report of Maeda *et al.* [59], together with the observations by De Saint-Jean *et al.* [51] and Tong *et al.* [58], emphasized the idea that a number of ORPs could mediate the non-vesicular transport of phospholipids such as PS, PI4P, and possibly PIP_2_ [37]. This hypothesis would be consistent with the earlier observations that yeast Osh proteins are not essential for ergosterol transport from the ER to the PM [39], and that a subset of these proteins (Osh1p, Osh3p, Osh6, Osh7p) display *in vitro* very weak sterol transfer activity [38]. The lipid ligands of human and yeast ORPs identified by structural analyses or ligand binding/transfer assays employing purified proteins are summarized in Table 1.

**Table 1:** Ligands identified for the ORDs of human and *Saccharomyces cerevisiae* ORPs by structural analyses or *in vitro* binding/transport assays.

Protein	ORD ligand(s)	Reference(s)
*Homo sapiens*		
OSBP	25OHC ^1^, 20OHC, 7KC ^2^, 22(R)OHC, 22(S)OHC, 7OHC, cholesterol + other oxysterols specified in [42]	[40,42,43,44]
ORP4/OSBP2	25OHC, 20OHC, 22(S)OHC, 7OHC, 7KC, 22(R)OHC, cholesterol	[43,61]
ORP1	24(S)OHC, 22(R)OHC, 7KC, 25OHC, cholesterol	[46,48]
ORP2	22(R)OHC, 7KC, 25OHC, cholesterol	[45,46]
ORP3	?	
ORP5	Dehydroergosterol, cholesterol?, PS ^3^	[49,59]
ORP6	?	
ORP7	?	
ORP8	25OHC?, cholesterol	[62,63]
ORP9	Cholesterol	[50]
ORP10	Cholesterol, PS	[47,59]
ORP11	?	
*Saccharomyces cerevisiae*		
Osh1p	Cholesterol?	[38]
Osh2p	Cholesterol	[38]
Osh3p	PI4P ^4^, cholesterol?	[38,58]
Osh4p	Ergosterol, cholesterol, 7OHC, 20OHC, 25OHC, dehydroergosterol, PI4P, PIP_2_? PS?	[33,37,51]
Osh5p	Cholesterol	[38]
Osh6p	PS	[59]
Osh7p	PS	[59]

^1^ hydroxycholesterol, ^2^ ketocholesterol, ^3^ phosphatidylserine, ^4^ phosphatidylinositol-4-phosphate.

## 4. Function of ORPs at Membrane Contact Sites

**Figure 3:**
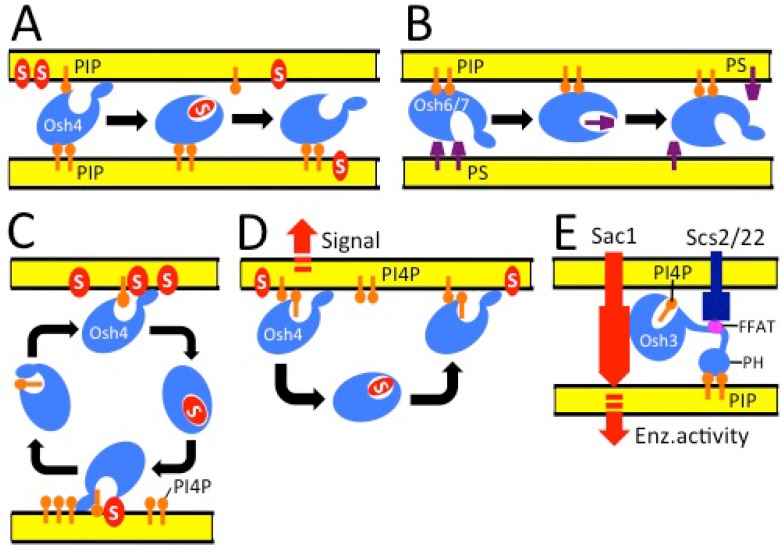
Models for the functions of yeast Osh proteins. (**A**) Osh4p acts at an ER-PM contact site, employs two membrane binding surfaces to associate with the bilayers via interactions with phosphoinositides (PIP) and other phospholipids, and transfers sterols (S) from one membrane to another [38]. (**B**) Osh6p and Osh7p transfer phosphatidylserine (PS) from the ER to the PM at membrane contact sites [59]. (**C**) Osh4p binds sterols (S) and phosphatidylinositol-4-phosphate (PI4P) in an exchange-type fashion within the same pocket, and transfers these two lipids in opposite directions [51]. (**D**) Osh4p binds to membranes of the *trans*-Golgi network and endosomes via interactions with PI4P. This association has a signaling function, and is negatively regulated by sterol binding to Osh4p, which detaches the protein from membranes [55]. (**E**) Osh3p acts at an ER-PM contact site, where it interacts with ER VAP proteins (Scs2p and -22p) through its two phenylalanines in an acidic tract (FFAT) motif and with PIPs via both its pleckstrin homology (PH) domain and the ORD ligand-binding tunnel, which accommodates PI4P. Osh3p controls the activity of the PI4P phosphatase Sac1p, an integral ER membrane protein, towards its substrate PI4P at the PM [58,67].

The capacity of most ORPs to target both the ER and other, non-ER organelle membranes, together with the observed localization of the yeast ORP Osh1p at a membrane contact site, the nucleus-vacuole junction [64,65], prompted the idea that these proteins could execute functions at MCSs [66]. This concept has gained increasing experimental support in both yeast and mammalian cells. Four yeast ORPs, Osh2p, Osh3p, Osh6p and Osh7p, were reported to be enriched at the cortical ER or ER-PM contacts [38,60]. Stefan *et al.* [67] provided convincing evidence that yeast Osh3p acts to recruit the PI4P phosphatase Sac1p anchored at ER membranes, at sites where it has access to its substrate PI4P on the PM. The data thus suggested a function of Osh3p as an organizer of functional protein complexes at ER-PM MCSs, with potential impacts on PM signal transduction and vesicle transport. In the light of the localization of Osh6p and Osh7p at ER-PM contact zones [38], the observations of Maeda *et al.* [59] on a functional role of these proteins in PS transport to the PM suggest that they could be part of a MCS-localized machinery responsible for flipping newly synthesized PS from the ER to the PM. The PM-associated ER domains in yeast are reported to have high PS synthetic activity [68]. One is therefore tempted to speculate that PS binding Osh proteins could be strategically located to couple PS synthesis to immediate transfer of this aminophospholipid to its destination, the cytoplasmic leaflet of the PM. Here, ORPs could function analogously to the ceramide transporter (CERT) which, according to accumulating evidence, acts at ER-*trans*-Golgi contact sites to flip ceramides accommodated within its START domain, to Golgi for sphingomyelin synthesis [69]. Models on the function of yeast Osh proteins are depicted in Figure 3.

Among the mammalian ORPs, the presence of determinants specifying both ER association and targeting to other organelles implicates putative functions at MCSs [36]. Factual evidence for a MCS-associated function has been reported for ORP1L, which interacts with ER VAP proteins through its FFAT motif and with the late endosomal (LE) small GTPase Rab7 via its N-terminal ankyrin repeat domain [70]. Upon cellular cholesterol depletion or expression of sterol-binding deficient mutant ORP1L, ORP1L-positive LE become scattered and display increased association with ER membranes, reflecting elevated affinity for the ER VAP proteins [48,71]. In contrast, knock-down of ORP1L increases the overall motility of LE, and mutation of the ORP1L FFAT motif mediating binding to VAPs in the ER has a similar effect [48]. When sterol is abundant, the sterol-bound conformation of ORP1L detaches from the ER and a tripartite complex of Rab7, ORP1L, and a second Rab7 effector, RILP (Rab7 interacting lysosomal protein) recruits dynein-dynactin motors on the LE surface, resulting in microtubule minus end-directed movement of LE towards the microtubule-organizing center [71,72]. The latest report provides evidence that ORP1L acts as a sterol-controlled switch regulating the interactions of RILP with dynactin and the homotypic vacuole fusion and protein sorting (HOPS) complex, which mediates organelle/transport vesicle docking on LE/lysosomes [73]. Thus, ORP1L controls the motility and subcellular distribution of LE as well as the docking/fusion of vesicles/organelles in the late endocytic pathway, with impacts on both protein transport and the cellular efflux of endocytosed cholesterol [48]. Of interest, and possibly related to the function of ORP1L, Du *et al.* [49] reported that ORP5, a protein integrally anchored to ER membranes, can be co-immunoprecipitated with the late endosomal cholesterol egress factor Niemann-Pick C1 (NPC1), and ORP5 knock-down resulted in accumulation of cholesterol in the limiting membrane of LE. The authors suggested that ORP5 might act at ER-LE contacts to mediate direct routing of endosomal cholesterol to the ER for esterification, a hypothesis awaiting further experimental dissection. They also provided *in vitro* evidence that the ORD of ORP5 is able to transfer cholesterol, indicating that the ligand-binding cavity of ORP5 may have the capacity to bind both sterol and PS [59].

## 5. Conclusions

The prevailing view has been that ORPs/OSBPLs function as sterol sensors relaying information to machineries responsible for different aspects of cell regulation, or as sterol transporters. We have envisioned that, via action as sterol and phosphoinositide sensors at organelle interfaces, ORPs modulate the lipid compositions of specific organelle membranes and the assembly of effector protein complexes, with impacts on signaling, vesicle transport, and lipid metabolism [36]. The report of Raychaudhuri *et al.* [37] produced the first indications suggesting that ORPs could, in addition to sterols, bind and transport glycerophospholipids. De Saint-Jean *et al.* [51] demonstrated that yeast Osh4p binds both sterols and PI4P, and can exchange the bound ligand. The authors provided evidence that Osh4p could transport the two lipids in opposite directions. However, the work of Mousley *et al.* [55] rather suggested that Osh4p binds to TGN and endosomal membranes via PI4P, with impacts on secretory membrane trafficking and metabolic control, and that this membrane association is negatively regulated by sterol binding. Recent work by Tong *et al.* [58] provided evidence that yeast Osh3p binds PI4P but not sterols, due to the limited dimensions of its ligand binding cavity, and that the ability to bind PI4P may be a property conserved among all or most ORPs. Maeda *et al.* [59] produced structural and functional evidence for the closely related Osh6p and Osh7p acting as PS transporters, and that also human ORP5 and 10 have the capacity to bind PS. This hallmark report supported the early findings of Raychaudhuri *et al.* [37] and the conclusion of Tong *et al.* [58] that the ORDs of ORPs are not confined to sterol liganding, but ORPs actually are LTPs with a wide spectrum of lipidous ligands, executing heterogeneous cellular functions. Thus, the term frequently used for the entire protein family, *oxysterol-binding proteins*, is a misnomer. While acting as sensors mediating lipid signals is definitely one aspect of ORP activity, their roles in controlling intracellular lipid fluxes and organelle lipid compositions should be addressed systematically, in order to reach a comprehensive understanding of this highly conserved protein family.
